# Regional impact of large-scale climate oscillations on ice out variability in New Brunswick and Maine

**DOI:** 10.7717/peerj.13741

**Published:** 2022-08-18

**Authors:** Carling R. Walsh, R. Timothy Patterson

**Affiliations:** Department of Earth Sciences, Carleton University, Ottawa, Ontario, Canada

**Keywords:** Lake ice out phenology, Climate teleconnections, Climate change, Time series analysis, Eastern North America

## Abstract

The available ice out (the date of disappearance of ice from a water body) records were analyzed from four relatively closely spaced lakes in southwestern New Brunswick (Harvey, Oromocto, Skiff) and eastern Maine (West Grand Lake), with the longest set of available observations being for Oromocto Lake starting in 1876. Results of a coherence analysis carried out on the ice out data from the four lakes indicates that there is regional coherence and correspondingly, that regional drivers influence ice out. These results also indicate that ice out dates for lakes from the region where records have not been kept can also be interpolated from these results. As the ice out record was coherent, further analysis was done for only Oromocto Lake on the basis of it having the longest ice out record. Cross-wavelet analysis was carried out between the ice out record and a variety of cyclic climate teleconnections and the sunspot record to identify which phenomena best explain the observed ice out trends. The most important observed contributors to ice out were the North Atlantic Oscillation (NAO) and the El Niño Southern Oscillation (ENSO), with observed periodicities at the interannual scale. At the decadal scale the Pacific Decadal Oscillation (PDO) and the 11-year solar cycle were the only patterns observed to significantly contribute to ice out.

## Introduction

Ice phenology data comprises a series of annual dates corresponding to when ice forms in the fall (ice in/on) and recedes in the spring (ice out/off) (Jensen et al., 2007; Latifovic & Pouliot, 2007; Patterson & Swindles, 2015; Imrit & Sharma, 2021). Ice in/out dates are considered to be influenced by precipitation, insolation, discharge, humidity, and most significantly, air temperature (Walsh et al., 1998; Futter, 2003; Livingstone et al., 2010; Patterson & Swindles, 2015; Imrit & Sharma, 2021). The variation in ice phenology and—by extension—the length of ice-free seasons can impact lake chemistry, productivity, and overall ecosystem health (Blenckner et al., 2007; Couture et al., 2015; Tan, Yao & Zhuang, 2018). For example, changes in ice out dates have a significant influence on the hydroecology of lakes with earlier ice out dates resulting in warmer spring water temperatures, increased light availability, and changes to circulation patterns (Hodgkins, 2013). Ensuing changes in spring and summer development of phytoplankton and zooplankton communities significantly impact the trophic structure of lake ecosystems (Blenckner, Omstedt & Rummukainen, 2002; Arhonditsis et al., 2004). As an example, the occurrence of potentially harmful cyanobacteria blooms, an emergent concern in this region, are significantly influenced by the length of the open water season in lakes (Ewing et al., 2020; Sivarajah et al., 2021). A full understanding of the dynamics of changes in ice out dates over time thus has important implications for determining the impact of climate variability on the hydrology, aquatic ecosystems, and a regional economy that relies heavily on lakes as an important recreational resource.

In many lakeside communities in North America and elsewhere, ice out dates are available from local lake associations and individual or groups of citizen scientists who have kept records for over a century in the interests of transportation, recreation, lotteries, or simply out of curiosity (Futter, 2003; Benson et al., 2012; Patterson & Swindles, 2015). Given their length—into the 19th century for many eastern North American lakes—it is possible to attempt to identify interannual, interdecadal, and multidecadal climatic drivers from these time series (Patterson & Swindles, 2015). Such an approach is useful when instrumental records are not available, or to complement data from nearby weather stations, which may not be as long or as complete. These records have been used previously to examine the interconnectivity of ice out and climate records, as well as to assess trends and cycles archived in these data sets. Strong links between ice out and winter air temperature have been noted, as well as the influences on ice out dates by a variety of large-scale climatic oscillations (*e.g.*, Quasi-Biennial Oscillation (QBO), North Atlantic Oscillation (NAO), El Niño Southern Oscillation (ENSO)) has been recognized (Livingstone, 2000; Magnuson, Benson & Kratz, 2004; Bonsal et al., 2006; Ghanbari et al., 2009; Livingstone et al., 2010; Bai et al., 2012; Mudelsee, 2012; Sharma et al., 2013; Fu & Yao, 2015; Patterson & Swindles, 2015; Imrit & Sharma, 2021). However, more robust time series analyses have not yet been widely carried out to verify these oscillations as the drivers of local or regional ice out patterns or to understand the time-varying relationship between ice out and the climate oscillations.

The purpose of this article is to use statistical and time series analysis techniques to: (1) determine the spatial coherence of lake ice out in four closely spaced inland lakes from southwestern New Brunswick, Canada and eastern Maine, USA; (2) provide a robust assessment of the periodic nature of the ice out records; and (3) assess and correlate potential drivers of the periodic changes in ice out dates. The trends and cycles documented for seven well-known climatic oscillations—the Schwabe Solar Cycle (SSC), Arctic Oscillation (AO), Atlantic Multidecadal Oscillation (AMO), Pacific Decadal Oscillation (PDO), Quasi-Biennial Oscillation (QBO), North Atlantic Oscillation (NAO), and El Niño Southern Oscillation (ENSO)—will be compared against year over year changes in lake ice out dates to assess their relationships. Each of these oscillations and their effects on the study region are described in Table 1. To assess the relationships between the non-linear lake ice out time series and large-scale climate oscillations, various spectral analyses were carried out. These methods allow for the conversion of time-domain data (*e.g.*, a time series) to its frequency-domain counterpart, revealing the cyclic components of a given signal. Spectral analysis techniques have been used in many climate and environmental studies to provide information on the periodic nature of various systems and improve the understanding of recurrent patterns. Recognizing periodic patterns in natural systems helps provide context for observed changes, particularly when assessing the impact of quasi-linear processes (*e.g.*, anthropogenic climate change) (Ng & Chan, 2012; Colombo, Magazù & Caccamo, 2018). Furthermore, demonstration that the ice out trends and cycles observed in the studied lakes are comparable is important as it would infer that limnological impacts influenced by ice out variability could be applied to lakes in the region where ice out data is not available. The understanding of lake ice out variability and its influencing factors could assist policy makers and local communities in the planning and preparation of lake-related activities and ecological monitoring in future seasons.

**Table 1:** Descriptions and regional effects of various climatic oscillations known to impact Atlantic Canada and United States.

**Climatic oscillation**	**Notation**	**Cycle length (years)**	**Description**	**Common impact on eastern North American climate**	**Citations**
Schwabe Solar Cycle	SSC	∼11, 8–17	An oscillation in the annual number of sunspots occurring, relating to total solar irradiance. The increases in total solar irradiance and UV irradiance during sunspot maxima drive dynamic changes in global stratospheric and tropospheric temperatures.	Increases in temperature during sunspot maxima, decreases in temperature during sunspot minima. Various links to precipitation and precipitation-related parameters.	Lassen & Friis-Christensen (1995), Rind et al. (2008), Lockwood (2012) and Hathaway (2015)
Atlantic Multidecadal Oscillation	AMO	∼64, 50–90, 16–24 subharmonics	An oscillation in the circulation pattern of warm and cool Atlantic ocean surface waters. Warm (AMO+) phases occurred from ∼1925–1965 and ∼1990-present, cool (AMO-) phases occurred from ∼1900–1925 and ∼1965–1990.	AMO+ is associated with increased temperatures, decreased precipitation, and greater drought probability.	Schlesinger & Ramankutty (1994), Enfield, Mestas-Nuñez & Trimble (2001), Knight, Folland & Scaife (2006), Dima & Lohmann (2007), Feng, Hu & Oglesby (2011), Knudsen et al. (2011), Ruiz-Barradas, Nigam & Kavvada (2013) and Alexander, Kilbourne & Nye (2014)
Pacific Decadal Oscillation	PDO	∼Interannual–multidecadal; strongest oscillations in the 15–25 year and 50–70 year bands	Characterized by fluctuations in sea-surface temperature; during PDO+ phases, the western mid-latitude Pacific cools, whereas eastern mid- and low-latitude Pacific warms. Interannual fluctuations in the PDO are linked to ENSO and Aleutian Low variability.	PDO+ phases are associated with decreased precipitation in the Great Lakes region and cooler temperatures southeastern North America. Opposite patterns occur during PDO-.	Trenberth (1990), Latif & Barnett (1994), Minobe (1997), Minobe (1999) and Mantua & Hare (2002)
North Atlantic Oscillation	NAO	Poorly defined, typically interannual–interdecadal	A localized oscillation in the sea level pressure differential between the Azores High and the Icelandic Low in the northern Atlantic Ocean.	NAO+ phases are typically associated with more moderate temperatures and wetter conditions in eastern North America, and drier, more extreme temperatures during NAO-phases.	Hurrell (1995), Hurrell, Kushnir & Visbeck (2001), Hurrell et al. (2003) and Olsen, Anderson & Knudsen (2012)
Arctic Oscillation	AO	Poorly defined, typically interannual–interdecadal	A broad oscillation in sea-level pressure in the Northern Hemisphere, occurring in an annular band around the northern mid-latitudes. During its positive phase, the AO supports a low-amplitude jet stream, during an AO-phase the jet-stream becomes a high-amplitude waveform. The localized NAO is a constituent of the broad scale AO.	Brings cool Arctic air to the mid-latitudes during AO+; cool Arctic airmasses travel further south into North America during AO-.	Deser (2000), Ambaum, Hoskins & Stephenson (2001), Rogers & McHugh (2002) and Li et al. (2017)
El Niño Southern Oscillation	ENSO	2–10	An oscillation characterized by the changes in sea surface temperatures in the tropical Pacific. Driven by the variation in strength of tropical trade winds—causing greater or weaker degrees in the upwelling of cool, deep ocean water during El Niño (ENSO+) and La Nina (ENSO-), respectively—this oscillation influences many regions of the world via various teleconnections.	Warmer, drier conditions during El Niño; cooler, wetter conditions during La Nina.	Philander (1983), Ropelewski & Halpert (1986), Philander (1989), Moy et al. (2002) and Hu & Feng (2012)
Quasi-Biennial Oscillation	QBO	2.1–2.4	The oscillation between westerly and easterly winds in the equatorial stratosphere. Air masses then propagate downward to the troposphere and are subsequently propagated poleward via teleconnections with surface waves.	Cooler temperatures during the westerly (QBO+) phase, warmer temperatures during the easterly (QBO-) phase.	Lindzen & Holton (1968), Dunkerton (1997), Baldwin et al. (2001), Chattopadhyay & Bhatla (2002), Sharma & Magnuson (2014), Patterson & Swindles (2015) and Gray et al. (2018)

## Methods

**Figure 1:**
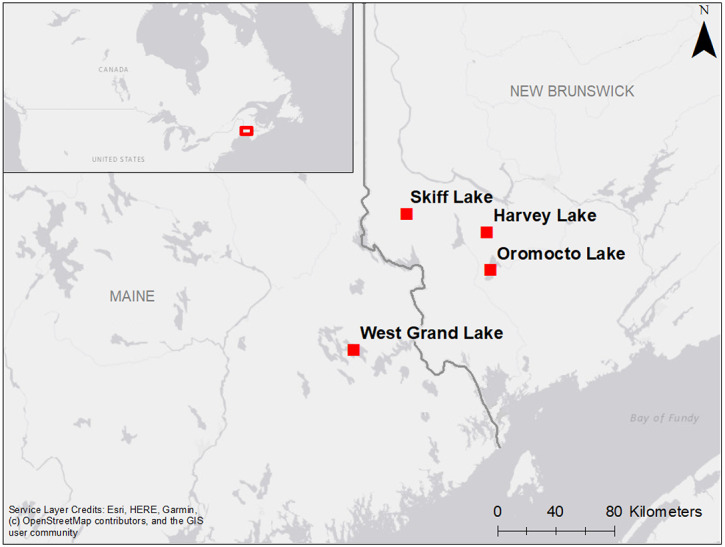
Location of lakes in New Brunswick and Maine for which ice out records were obtained.

Ice out records from three southwest New Brunswick lakes (Oromocto Lake (1876–2021), Skiff Lake (1933–2021), and Harvey Lake (1975–2021)) and one inland eastern Maine lake (West Grand Lake, 1878–2021), all located within 75 km of each other (Fig. 1), were obtained from local lake associations and the Maine Department of Agriculture, Conservation and Forestry (Article S1). While definitions of ice out may vary slightly between each lake and its ice out observers, ice out is generally defined as the first day that a lake is navigable with no impedance from ice cover. Depending on the ice out observers, criteria may also include the disappearance of ice from major coves (Article S1).

All data analysis was completed using Matlab. Each ice out record was checked for normality and detrended prior to applying any spectral analysis techniques. The Julian date of recorded ice out was used in each of the analyses completed. These dates are relative to each lake. As the patterns in the ice out records are the points of interest in this study, this relative data did not require standardization. The Harvey, Skiff, and West Grand lakes ice out records were each, in turn, compared to the Oromocto Lake record using wavelet coherence (WTC) and Pearson’s correlation coefficients to assess spatial relationships within the study region. Oromocto Lake was chosen for this role due to it having, by two years, the longest ice out record of the four lakes. WTC transformations were carried out as they have been demonstrated to reliably identify local correlations between oscillating time series (Grinsted, Moore & Jevrejeva, 2004). The use of WTCs build upon linear correlation, as information about the covariance of two time series and their intermittent and time-varying correlations are provided. These results are presented by a series of arrows on a time-frequency spectrum, where the direction of the arrows indicates the relative phase of the two time series at a given time-frequency coordinate. Arrows pointing to the right (left) indicate that the time series are in-phase (anti-phase), whereas downward (upward) arrows indicate that one signal is being led by (lagged by) the other. In this case, downward (upward) arrows indicate that the Oromocto Lake record is oscillating ahead of (behind) the respective lake record it is being compared to. A strong coherence and correlation between the lake ice out records are indicative of regional trends in ice out and, by extension, climate, whereas weak correlations indicate more climatic variation in the Atlantic region.

To assess long-term trends and fluctuations, locally weighted linear regressions (LOWESS) was carried out for each of the four lake ice out records over 20-year periods (Cleveland, 1979; Cleveland, 1981). The 95% confidence intervals on the LOWESS models were calculated by bootstrapping without replacement (1,000 replicates).

Due to strong correlation and coherence between each of West Grand, Harvey, and Skiff lakes to Oromocto Lake, only the ice out record for Oromocto Lake was assessed for its periodic components (for simplicity) using spectral and wavelet analysis. Thomson’s Multitaper Method (MTM, Thomson, 1982; Dorothée, 2020) was used to transform the time series data into its corresponding frequency domain by comparing various sine functions of different amplitudes and wavelengths to the time series. High correlation of a given sine curve to the input time series results in higher spectral power at the corresponding frequency of the sine curve, which is shown on one axis of the spectral analysis plots. Frequency is shown on the other axis. With this method, one can discern discrete frequency information of the input time series by examining the peaks of the resulting periodogram. Furthermore, the MTM uses multiple ‘tapers’, unlike other nonparametric spectral analysis methods. Tapers, or windows, are symmetric, zero-sum curves that are pairwise orthogonal to the input time series. A time series can be multiplied by a taper to minimize the spectral leakage of the signal. The MTM uses a set of idealized tapers to transform the input time series before comparing it to the various sine curves. The spectra of all tapered time series data are then averaged, ultimately resulting in a more stable and robust spectral estimate (Schulz & Mudelsee, 2002; Husson et al., 2014).

The mean spectral estimate was compared to 80, 90, 95, and 99% false alarm levels, calculated using chi-square confidence estimate, and the red noise (AR1) model, calculated using a Monte Carlo loop (1,000 replicates) (Schulz & Mudelsee, 2002; Husson et al., 2014; Dorothée, 2020).

Continuous wavelet transforms (CWTs) were used in tandem with the MTM to assess the time-frequency variation of the time series. Similar to the MTM, CWTs compare a waveform to a time series, the results of which present the spectral information of a time series; this method differs from the MTM, as it does not provide discrete spectral peaks, though it does indicate the variation in these signals in time (Torrence & Compo, 1998; Grinsted, Moore & Jevrejeva, 2004). This time-varying spectral estimate is computed by comparing a waveform (in this case, the Morlet wavelet) at a range of frequencies and amplitudes to the time series throughout a sequence of time steps. The correlation of the given waveform at each time step results in a spectral estimate unique to that moment in time. This time-varying frequency estimate is presented in three axes: an *x*-axis indicating the time, a *y*-axis indicating the frequency or period, and a colour axis representing the spectral power. The 95% confidence interval was calculated using the chi-square estimate (Torrence & Compo, 1998) and is shown as a solid black line on the resulting wavelet scalograms. Cones of influence are also depicted on the scalograms; these cones indicate the time-frequency information on a given scalogram that may be subject to ‘edge effects’ from the boundaries of the time series. Using both the MTM and CWT in tandem provide both discrete and continuous frequency domain data.

The Oromocto Lake ice out record was then compared to each of the SSC (SILSO, 2021), PDO (Deser et al., 2016), AMO (Trenberth et al., 2021), AO (NCAR, 2020), NAO (Hurrell & NCAR, 2020), ENSO (Trenberth & NCAR, 2020), and QBO (NCAR, 2013) *via* cross wavelet transforms (XWTs). XWTs are a type of spectral transform similar to a CWT, in that the time-varying frequency information of time series is computed. However, the purpose of an XWT is not to present only this time-frequency information, but rather to compare a reference signal with an analyte signal to assess the common time-frequency components to the two signals. The resulting XWT scalogram appears the same as that of a CWT, though only the common elements time-frequency spectrum is displayed (Grinsted, Moore & Jevrejeva, 2004). Using this method, we can assess the relationships between the reference signal (*i.e.,* Oromocto Lake ice out) and each analyte signal (*e.g.*, the AMO or the PDO).

## Results

### General trends in lake ice phenology

**Figure 2:**
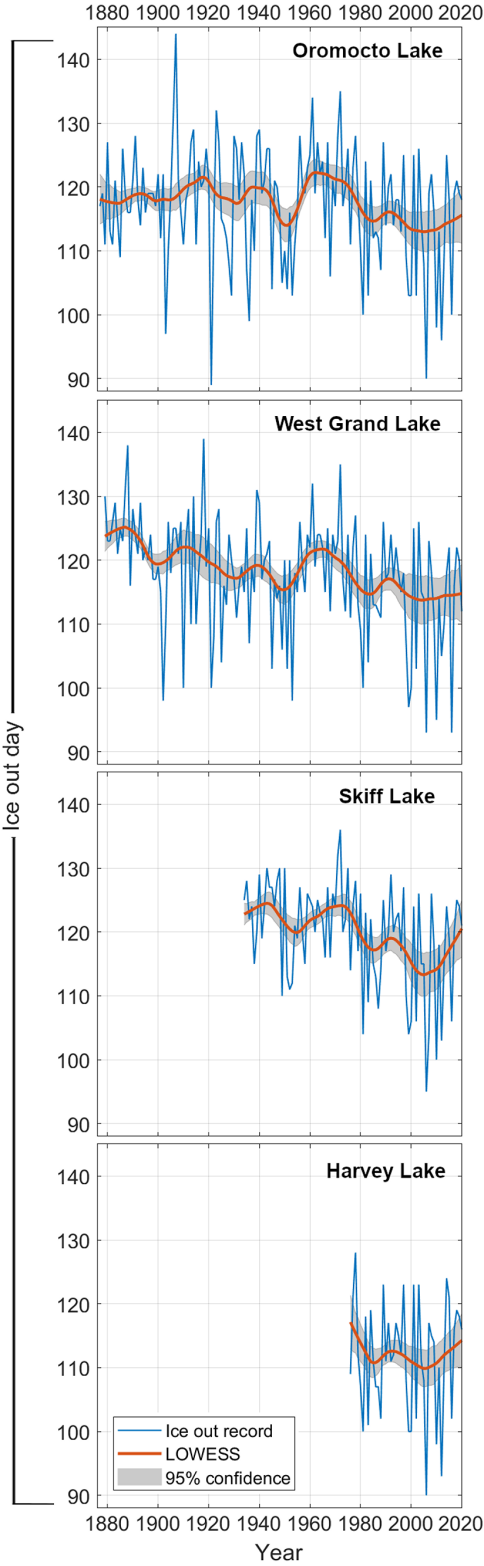
LOWESS regressions (span—20 years, ± 95% confidence) for each lake ice out record for their available time frames.

While ice out dates can fluctuate by as much as 20 + days year over year, long-term averages in lake ice phenology are generally more consistent, only varying up to 10 days decade over decade (Fig. 2). On West Grand Lake, ice persisted latest in the spring on average from ∼1880–1890 before trending to earlier ice out dates until ∼1900, shifting later in the spring again until ∼1910. In contrast, average ice out dates for Oromocto Lake were relatively consistent from 1876–1910. From the mid to late 1910s onward, ice out on both West Grand Lake and Oromocto Lake shifted earlier in the year until the 1930s. Average ice out dates shifted later again through the next decade until the early 1940s on each of West Grand Lake, Oromocto Lake, and Skiff Lake before becoming earlier around 1950. These three lakes each exhibit a later ice out peak, spanning the entire record in the 1960s and 1970s before generally declining towards 2000 (Harvey Lake also exhibits this decline). Since the early 2000s, average ice out dates have generally shifted later in the year on all lakes except West Grand Lake. West Grand Lake ice out indicates the most substantial change between the late 19th century and the early 21st century, with the average ice out date lowering from approximately Julian Day 124 to Julian Day 115. Oromocto Lake does not show this same change, with average late 19th century ice out occurring prior to Julian Day 120 and early 21st century ice out occurring around Julian Day 115.

### Lake ice out coherence and correlation

**Figure 3:**
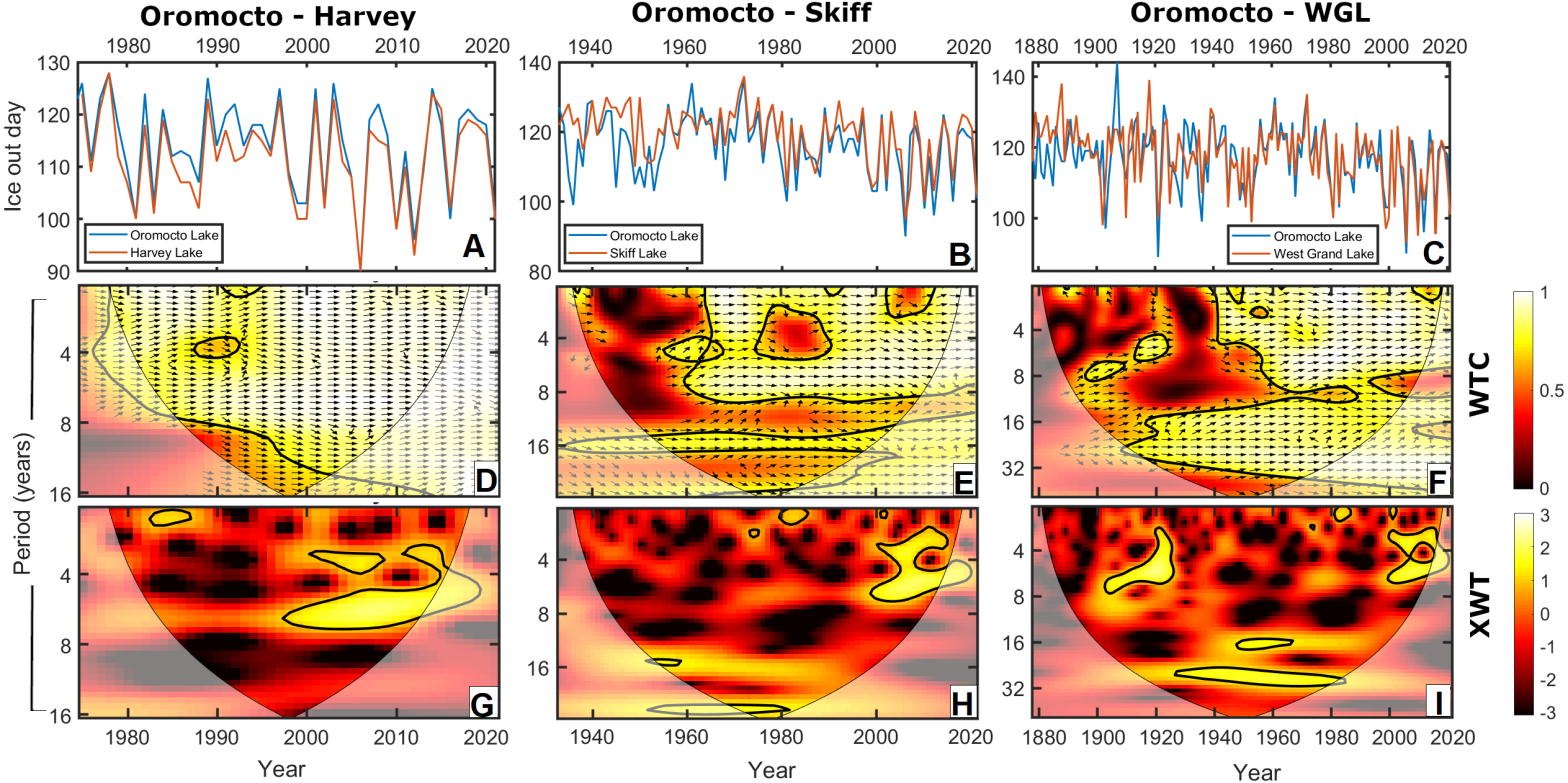
(A–C) Graphical comparisons, (D–F) wavelet coherence (WTC), and (G–I) cross wavelet transforms (XWTs) between the Oromocto Lake ice out record and each of the Harvey Lake, Skiff Lake, and West Grand Lake ice out records during relative time intervals. Arrows on the wavelet coherence scalograms indicate locally phase locked behaviour, where right-pointing arrows indicate in-phase relationships, left-pointing arrows indicate anti-phase relationships, and up/down-pointing arrows indicate lagging/leading relationships.

**Figure 4:**
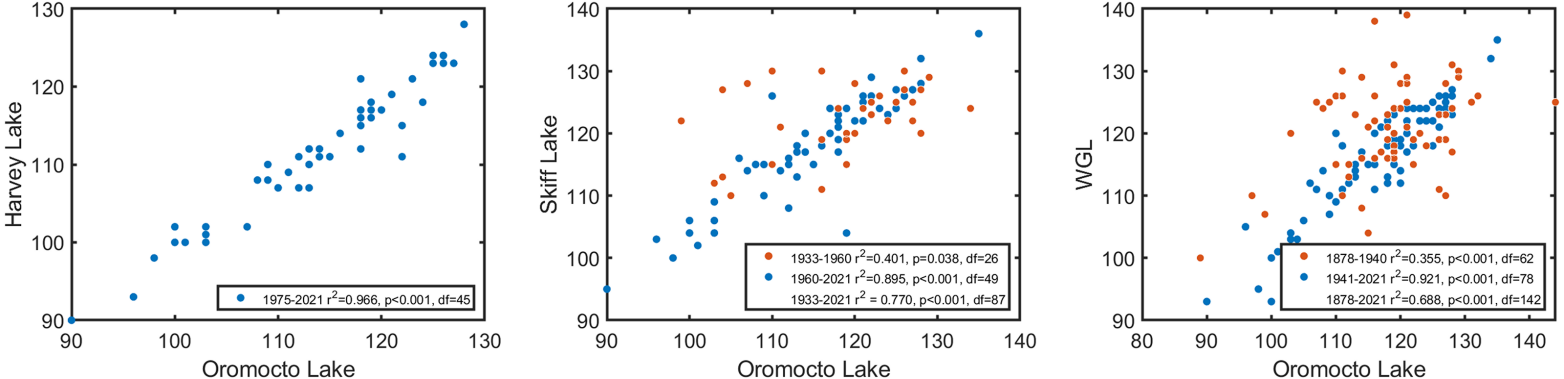
Correlations between the Oromocto Lake ice out record and each of the Harvey Lake, Skiff Lake, and West Grand Lake ice out records. Pearson’s correlation coefficients and *p*-values are given. Multiple correlations for Oromocto Lake with each of Skiff and West Grand Lakes are given as these correlations strengthen during later time intervals.

Oromocto Lake exhibited very strong coherence with each of the three other lakes in this study, particularly in the latter 20th century. The coherence with each lake spans interannual to interdecadal bands at the 95% confidence level (Figs. 3D, 3E and 3F), though notable non-significant coherence bands include ∼10 years (all lakes), ∼21 years (Skiff Lake), and ∼42 years (West Grand Lake). West Grand Lake has the most comparable record length to Oromocto. Prior to ∼1940, these records have less coherence and only a moderate correlation (*r*^2^ = 0.355, *p* = 0.004, *df* = 62), but are strongly coherent and correlated afterwards (*r*^2^ = 0.921, *p* < 0.001, *df* = 78; Figs. 3F and 4). The Skiff Lake record is similar in that correlation was initially lower (*r*^2^ = 0.401, *p* = 0.038, *df* = 26) with coherence primarily at interdecadal scales but increased after 1960 (Fig. 3E), whereafter the records are coherent and strongly correlated (*r*^2^ = 0.895, *p* < 0.001, *df* = 49). Harvey Lake has the shortest available ice out record and shows strong and significant coherence (Fig. 3D) and correlation with Oromocto Lake over the available dates (*r*^2^ = 0.966, *p* < 0.001, *df* = 45, Fig. 4). The strong overall correlation and coherence of all three lakes with Oromocto Lake ice out justifies the use of the Oromocto Lake record as a representative sample for the region, particularly for the mid-20th century to present-day interval.

### Periodicity in lake ice out

**Figure 5:**
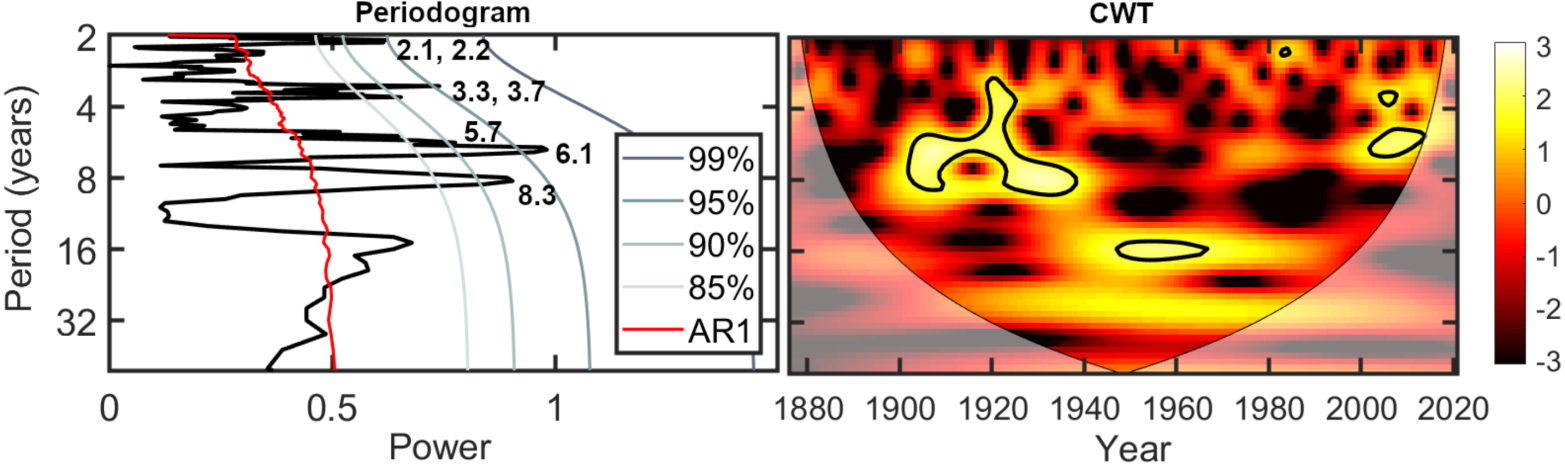
Spectral analysis and the continuous wavelet transform (CWT) for the Oromocto Lake ice out record, 1876–2021.

Figure 5 shows a periodogram and a continuous wavelet transform for the ice out record from Oromocto Lake. The time series is primarily characterized by interannual oscillations with periods ranging from 2.1 to 8.3 years. These oscillations are most significantly (>95% confidence) concentrated between approximately 1900–1940 and 2000–2015, but they also occur intermittently with lower significance throughout the ice out record, as recognized in both the MTM periodogram and the CWT. Some interdecadal oscillations are also present at a lower significance; ∼9–18 year oscillations persist from approximately 1940–1990 (significant at the 95% confidence level from approximately the mid-1940s to ∼1970), as well as a ∼23–34 year oscillation from 1920–2000.

**Figure 6:**
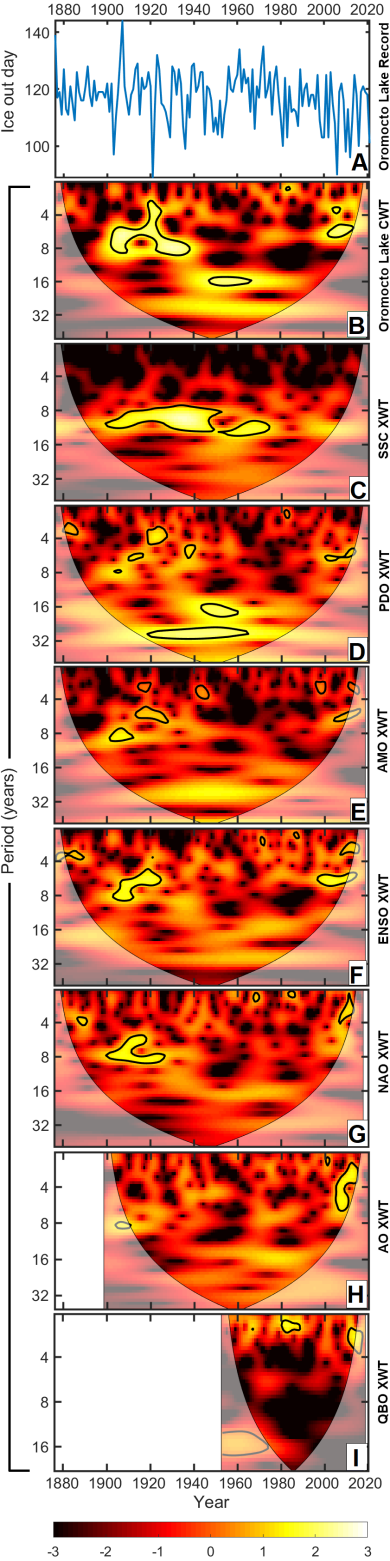
For Oromocto Lake ice out, (A) graphical representation, (B) the continuous wavelet transform (CWT), and (C–I) the cross wavelet transforms (XWTs) between Oromocto Lake ice out and each of the Schwabe Solar Cycle (SSC), Pacific Decadal Oscillation (PDO), Atlantic Multidecadal Oscillation (AMO), El Niño Southern Oscillation (ENSO), North Atlantic Oscillation (NAO), Arctic Oscillation (AO), and Quasi-Biennial Oscillation (QBO).

The XWTs between the Oromocto Lake ice out time series and the records for seven known regional climate oscillations are provided in Fig. 6, while the XWTs for Harvey, Skiff, and West Grand lakes where observed large-scale climate oscillations can be viewed in Fig. S1. A comparison of the XWT results with the CWT and periodogram helps clarify which climatic oscillations best explain observed oscillations in the ice out record.

At the interdecadal to multidecadal scales, the PDO, SSC, and AMO are of particular interest. The PDO shows strong relationships with cycles within the 15–18-year band (most significantly from ∼1940–1960) as well as a 23–34-year oscillation from 1920–1965. The SSC is linked to ∼7–13-year oscillations in the ice out dates, most significantly from 1900–1980. Less significant relationships exist during times of weak quasi-decadal oscillations in ice out (*e.g.*, 1995–2015). While the relationship for AMO is less significant (<95% confidence), there nevertheless exists an area of high incidence between the AMO and the ice out time series from approximately 1920 and 1980, with oscillations of 23–30 years. Furthermore, the beginnings of a 45+ year oscillation related to the AMO is just visible at the bottom of the AMO—ice out scalogram, indicating that the longer band of the AMO may have a relationship with ice out dates.

The most prevalent oscillations at the interannual scale are primarily associated with ENSO, NAO, AO, and QBO (Figs. 6F–6I). Both ENSO and NAO appear to have the strongest relationship with the interannual oscillations occurring between 1900–1920 (∼5–8-year oscillations). The AO exhibits a weaker relationship at this time-frequency location, though this may be influenced by the AO’s shorter temporal record. Each of these three climatic oscillations also exhibit significant relationships with the ice out record from approximately 2000–2015, in the 3–6-year return time range. The QBO primarily exhibits significant cyclic relationships with the ice out record at a period of 2.1–3.1 years (*e.g.*, ∼1966–1968, 1980–1990, Fig. 6I). A 12–18-year common oscillation is also present between ice out and the QBO from approximately 1953–1975. While they are usually associated with interdecadal to multidecadal oscillations, the PDO and AMO also show some instances of significant (>95% confidence) relationships, primarily in the 3–8-year return time bands during the time intervals spanning 1900–1940 and 2000–2015.

## Discussion

### Regional coherence and long-term patterns in ice out

The strong coherence and correlation exhibited between lake ice out records between closely spaced Harvey, Oromocto, Skiff and West Grand lakes (Figs. 3 and 4), and the similarity in long-term observed trends (Fig. 2) are indicative of regionally consistent patterns in ice out and ice out forcings. While localized factors such as the surrounding landscape, lake basin and shoreline characteristics, and wind exposure may influence some minor variation between ice out dates of these four lakes, the main drivers of ice out appear active at a regional scale. The long-term patterns in lake ice out in these lakes suggests only limited changes in year over year (20 + days) ice out variation since the late 19th century. While several shifts towards earlier or later average ice out have been recognized, a substantial change in average ice out since the late 19th was not strongly apparent (late 19th century average ice out occurred around Julian Day 120 (125) on Oromocto (West Grand) Lake whereas early 21st century ice out occurred around Julian Day 115 (115)). Rather, interdecadal shifts in ice out dates dominate the long-term variation, culminating in a shift toward later average ice out beginning in the early 2000s and continuing to present.

The general long-term pattern in lake ice out closely resembles the findings of Hodgkins, James & Huntington (2002) for ice out on northern New England lakes(which are latitudinally similar to the four lakes examined here) between the late 19th century to the year 2000. Hodgkins, James & Huntington (2002) note that lakes in the northern and mountainous regions of New England did not exhibit as strong of shifts toward earlier ice out dates in this time frame as lakes of southern New England did. The authors suggest that greater snow cover in the northern region decreases the sensitivity of lake ice cover to late winter regional air temperature, which is a primary driver of ice out. For the four lakes examined here, a similar moderation of ice out day may be occurring due to snow cover, subsequently decreasing the correlation to air temperature.

Prior to the mid-20th century, coherence and correlation between time series were lower though, for which a possible, but not confirmed explanation, may be related to a change in reporting methodology. For example, at Oromocto Lake, reporting of ice out dates have for decades been documented by citizen scientist Clayton Piercy who took over the duty of collating and recording ice out dates as a teenager in the 1940s. He used a single set of criteria (ice out declared when ice has disappeared from all coves) from the mid 20th century until his recent passing. The reporting of ice out on Oromocto Lake is now carried out collaboratively by many members of the Oromocto Lake Association using the same criteria as used by Clayton Piercy, but now aided by real time simultaneous social media monitoring of the entire lake. Prior to the mid 20th century, reports were made by many people at different locations on the lake, where based on examination of the data, ice out may have been declared when ice disappeared from the main body of the lake, and not slightly later when it disappeared from coves (Article S1). This unconfirmed hypothesis may explain the reduced coherence between the ice out record for Oromocto Lake and that from West Grand Lake through the earlier part of the ice out record.

### Interannual variations

Ice out in the study area is primarily dominated by interannual oscillations in the ∼2–8 year range (Figs. 3 and 5). A correlation with these periodicities is most apparent in the scalograms with ENSO and NAO (Fig. 6). These oscillations have also previously been related to variations in temperature and precipitation in the region (Shabbar, Bonsal & Khandekar, 1997; Shabbar, 2006; Toniazzo & Scaife, 2006; Bonsal & Shabbar, 2011; Zhao et al., 2013; Patterson & Swindles, 2015; Nalley et al., 2019). The effects of ENSO and NAO on ice out are similar to one another, for which there are two main explanations. The simplest explanation is that the dominant frequencies of the two oscillations are similar. Both oscillations have a particularly strong 4–8 year band (Rasmusson & Carpenter, 1982; Kestin et al., 1998; Setoh et al., 1999; Pozo-Vázquez et al., 2001; Barbosa, Silva & Fernandes, 2006; Olsen, Anderson & Knudsen, 2012), which is abundant in the ice out time series. The alternate explanation is that there is a nonstationary relationship between the ENSO and NAO, in which the two climatic oscillations interact and covary depending on their phases (Zanchettin et al., 2008; Seager et al., 2010; Zhang et al., 2019). These interactions may amplify late winter/early spring temperature and precipitation anomalies depending on the state of the oscillations’ nonstationary relationship, each of which are correlated to ice out dates (Hodgkins, James & Huntington, 2002; Hewitt et al., 2018).

The AO exhibited a relationship to ice out similar to that of the ENSO and NAO, though it was generally weaker (∼8 year common oscillations, ∼1910; 3–8 year common oscillations, ∼2000–2015). The NAO is a geographically localized subset of the AO with both being products of oscillations in atmospheric pressure which, in turn, impact atmospheric circulation and the movement of airmasses (Thompson & Wallace, 1998; Ambaum, Hoskins & Stephenson, 2001; Rogers & McHugh, 2002; Thompson & Lorenz, 2004; Cohen & Barlow, 2005). While the AO has been found to influence ice out and other climate variables elsewhere in eastern North America (Bonsal et al., 2006; Wang et al., 2010; Mishra et al., 2011; Wang et al., 2012; Imrit & Sharma, 2021), its weaker influence on Oromocto Lake ice out may be a result of the AO’s broader geographic expanse, in comparison to the NAO.

The QBO exhibited a relationship to observed ∼2–3 year oscillations in ice out, which suggests that it plays a role in driving interannual ice out variability, albeit less importantly than for the ENSO and NAO though. The QBO has previously been attributed as the driver of ∼2-year variations in ice out along the east coast of North America (Sharma & Magnuson, 2014; Patterson & Swindles, 2015; Imrit & Sharma, 2021), and has been related to other climatic variations, such as those in temperature or precipitation (Lau & Sheu, 1988; Baldwin et al., 2001; Chattopadhyay & Bhatla, 2002; Becker et al., 2008; Cherenkova, Bardin & Zolotokrylin, 2015). The QBO is linked to the strengthening/weakening and the overall stability of the Arctic polar vortex. The strength and stability of the polar vortex impacts the movements of large-scale airmasses, thus impacting mid-latitude temperatures, strom tracks, and precipitation (Holton & Tan, 1980; Walter & Graf, 2005; Anstey & Shepherd, 2014; Overland & Wang, 2019).

### Interdecadal variations

Interdecadal oscillations in ice out in these lakes were primarily correlated to the SSC and the PDO. While the quasi-decadal signal in the ice out time series was generally weak, it has a strong relationship to the SSC. This result suggests that while quasi-decadal oscillations are not always prominent in the ice out record, in the instances when they are present, they are primarily driven by the SSC. A strong relationship between the SSC and quasi-decadal oscillations has been previously observed for both ice out records (Sharma et al., 2013; Sharma & Magnuson, 2014; Fu & Yao, 2015; Hewitt et al., 2018) and temperature and precipitation records (Vines, 1984; Currie & O’Brien, 1988; Currie, 1993; Van Loon & Shea, 1999; Ogurtsov et al., 2008; Lockwood, 2012; Laurenz, Lüdecke & Lüning, 2019; Walsh & Patterson, 2022; Walsh & Patterson, 2022) due to its relation to total solar irradiance, which subsequently influences atmospheric, oceanic, and terrestrial heating (Yang et al., 2010; Kopp, 2014).

The PDO appears to be the primary driver correlated to 15–18 year and 23–34 year oscillations in ice out record, which is largely consistent with the prominent 15–25 year band of the PDO (Minobe, 1997; Minobe, 1999; Mantua & Hare, 2002; D’Orgeville & Peltier, 2007; Wu et al., 2011). Similar to what was observed with the quasi-decadal oscillations, these oscillations are not very prominent in the ice out record, but they have a strong relationship with the PDO when they are present. The correlation of the PDO to ice out is strongest prior to the 1977 PDO regime shift. Other PDO regime shifts (*e.g.*, 1890, 1925, and 1947) are somewhat recognizable in the PDO–ice out relationships (Fig. 6), though not amongst the statistically significant correlations. PDO regime shifts, particularly the 1976–1977 “Great Regime Shift” from PDO+ to PDO-, have been recognized as contributors to abrupt changes in climate and ecological systems worldwide (Hare & Mantua, 2000; Scheffer et al., 2001; Litzow, 2006; Le Goff et al., 2007; Wang et al., 2014; Jacques-Coper & Garreaud, 2015), and have been noted for influencing ice phenology, primarily *via* atmospheric temperature changes (Magnuson, Benson & Kratz, 2004; Bonsal et al., 2006; Ghanbari et al., 2009; Mishra et al., 2011; Wang et al., 2018).

The AMO also exhibited a correlation to the 23–34 year band in ice out, but not at a 95% confidence level. The observed AMO–ice out relationship is similar to that of the PDO, though weaker overall, exhibiting a statistically insignificant correlation to the 23–34 year band in ice out. As the AMO influences Atlantic climate events and patterns and is characterized by sea surface temperature variation, a relationship to east coast ice out variation is not unreasonable (McCabe, Palecki & Betancourt, 2004; Alexander, Kilbourne & Nye, 2014; Wang et al., 2018; Abiy, Melesse & Abtew, 2019). This weaker but persistent AMO–ice out relationship could also be related to the PDO, as it mirrors the PDO’s relationship to 23–34 year oscillations in ice out. There exists a moderate correlation between the indices for the AMO and PDO (McCabe, Palecki & Betancourt, 2004; D’Orgeville & Peltier, 2007; Wu et al., 2011; Ogunjo & Fuwape, 2020; Zhang et al., 2020); thus, the similarity in their influence on ice out variation may stem from mutual interactions.

Furthermore, the beginnings of a 45+ year AMO relationship to Oromocto Lake ice out were exhibited in Fig. 6. This provides a preliminary indication that the primary multidecadal mode of the AMO likely influences ice out in the New Brunswick and Maine region; however, further validating this relationship with the methods used here is not possible due to the limited time frame of ice out data available. A longer ice out record is needed to robustly assess the common multidecadal signals of the AMO (50–90 years) and regional ice out.

It should be noted that the interdecadal oscillations are most strongly recognized in the ice out record and most strongly correlated to other oscillations within the cone of influence on each CWT or XWT. While this may be an accurate representation of the time series, it is also possible that the concentration of these interdecadal oscillations within the cone of influence could be attributed to edge effects, where the frequency information of the input signal(s) may be distorted outside of the cone of influence (Grinsted, Moore & Jevrejeva, 2004). The CWT and XWT techniques are limited in this sense. The timing of low frequency oscillations cannot be conclusively determined without longer data records.

Lastly, a brief instance of an interdecadal-scale relationship was depicted between ice out and the QBO between approximately 1953–1980. This result likely stems from a modulatory effect between the SSC and the QBO, reported in several studies (Quiroz, 1981; Hamilton, 2002; Fischer & Tung, 2008), which has not persisted since the early 1980s.

## Conclusions

This study examined four inland lakes, all within 75 km of each other in eastern Maine and southwest New Brunswick, to assess regional drivers of ice out variability. The ice out records of three of these lakes (West Grand Lake in Maine, and Skiff Lake and Harvey Lake in New Brunswick) were first compared to that of Oromocto Lake in New Brunswick, as it had the longest record, to assess the presence of regionally consistent patterns. The lakes in the region were strongly correlated and coherent with Oromocto Lake’s ice out record, particularly from the mid-20th century, suggesting regional consistency in ice out patterns and their drivers. Inconsistencies prior to the mid-20th century may be attributed to changes in data-gathering methodology and personnel, though this hypothesis has not been confirmed.

Oromocto Lake ice out was then compared to various climatic oscillations *via* spectral, CWT, and XWT analysis. These time-series analysis techniques suggest that most ice out variation occurs primarily at the interannual scale. These variations are generally related to the ENSO and the NAO, particularly for periods of 4–8 years. Lower interannual oscillations (2–3 years), while less prominent in the ice out time series, are related to the QBO. Interdecadal to multidecadal variations form a secondary component of the ice out time series, and are most strongly driven by the SSC (quasi-decadal) and the PDO (15–25 year). A good understanding of the role of trends and cycles of regional climate drivers on ice out allows for the prediction of both future variation in regional lake ice out and regional climate. This is of importance as this data will inform policy makers and planners when considering future recreation, economic development, and environmental practices as they relate to adapting to changes in regional climate. Of particular environmental concern is that the length of the open water season is driven by ice out, which in turn significantly impacts lake ecosystems, particularly the significant emergent environmental threat to the region, cyanobacteria blooms.

##  Supplemental Information

10.7717/peerj.13741/supp-1 Article S1 List of lakes used in study, including geographic location, elevation, duration, and source

10.7717/peerj.13741/supp-2 Data S1 Annual ice out day for Oromocto Lake (1876–2021), Harvey Lake (1975–2021), Skiff Lake (1933-2021), and West Grand Lake (1878–2021)

10.7717/peerj.13741/supp-3 Figure S1 Graphical representation and the continuous wavelet transform (CWT) for Oromocto Lake Cross wavelet transforms (XWTs) between lake ice out records and each of the SSC, PDO, AMO, ENSO, NAO, AO, and QBO. Time scales all span 1876–2020 for easy comparisons.

## Additional Information and Declarations

### Data Availability

The following information was supplied regarding data availability: The raw annual ice out data is available in the Supplemental Files.
