# Supplementary material for: Regional impact of large-scale climate oscillations on ice out variability in New Brunswick and Maine
Source: PeerJ. 2022 Aug 18;10:e13741. doi: 10.7717/peerj.13741 (PMC9393007; doi:10.7717/peerj.13741)
Supplement: Article S1 [file peerj-10-13741-s001.pdf]

Article S1: List of lakes used in study, including geographic location, elevation, duration, and source.

Harvey Lake, NB, Canada
Lat/Long: 45.74368°, -67.03123°
Elevation: 146 m
Duration of Ice off record: 1975-2021
Ice out observers:
Record obtained from: Harvey Lake Association
https://sites.google.com/site/harveylakeassociation/

Skiff Lake, NB, Canada
Lat/Long: 45.824183°, -67.541774°
Elevation: 213 m
Duration of Ice off record: 1933-2021
Ice out observers: Harley Carr (1933-1952), Bill Crossley (1953-1961), Gerald Lochart (1961-1972), Al and Gar Conklin (1973-2013)
Record obtained from: Wikipedia http://en.wikipedia.org/wiki/Skiff_Lake_(New_Brunswick)

Oromocto Lake, NB, Canada
Lat/Long: 45.586134°, -67.011411°
Elevation: 107 m
Duration of Ice off record: 1876-2021
Ice off observers: Samuel McFarland; William Messer, sons John and Thomas Messer and grandson Henry Messer; John Rutherford and son John M. Rutherford; most recently Clayton Piercy (1944-2013) Record obtained from: Clayton Piercy and Oromocto Lake Association
https://www.facebook.com/oromoctolake

Notes: Ice-out has traditionally been determined as the day when the various coves of the lake are open to navigation, which may be several days later than when the main body of the lake is open to navigation. Clayton Piercy compiled the earlier portion of his Oromocto Lake record by visiting farms located along the western shore of Oromocto Lake where the ice out dates were variously written on barn doors, dairy cow stalls, woodshed walls and cellar ways. The records were remarkably coherent as all observers followed the same criteria for ice out across multiple generations (Clayton Piercy, pers. Comm. Dec. 2013).

West Grand Lake, ME, USA
Lat/Long: 45.234944°, -67.842491°
Elevation: 91 m
Duration of Ice off record: 1878-2021
Ice out observers: Marion Staples and family (three generations).
Record to 2008 from Hodgkins (2010). Record from 2009-2013 from: Maine Department of Agriculture, Conservation and Forestry
http://www.maine.gov/dacf/parks/water_activities/boating/ice_out_dates.shtml
