# Supplementary material for: Regional impact of large-scale climate oscillations on ice out variability in New Brunswick and Maine
Source: PeerJ. 2022 Aug 18;10:e13741. doi: 10.7717/peerj.13741 (PMC9393007; doi:10.7717/peerj.13741)
Supplement: Figure S1 — Cross wavelet transforms (XWTs) between lake ice out records and each of the SSC, PDO, AMO, ENSO, NAO, AO, and QBO. Time scales all span 1876–2020 for easy comparisons. [file peerj-10-13741-s003.pdf]

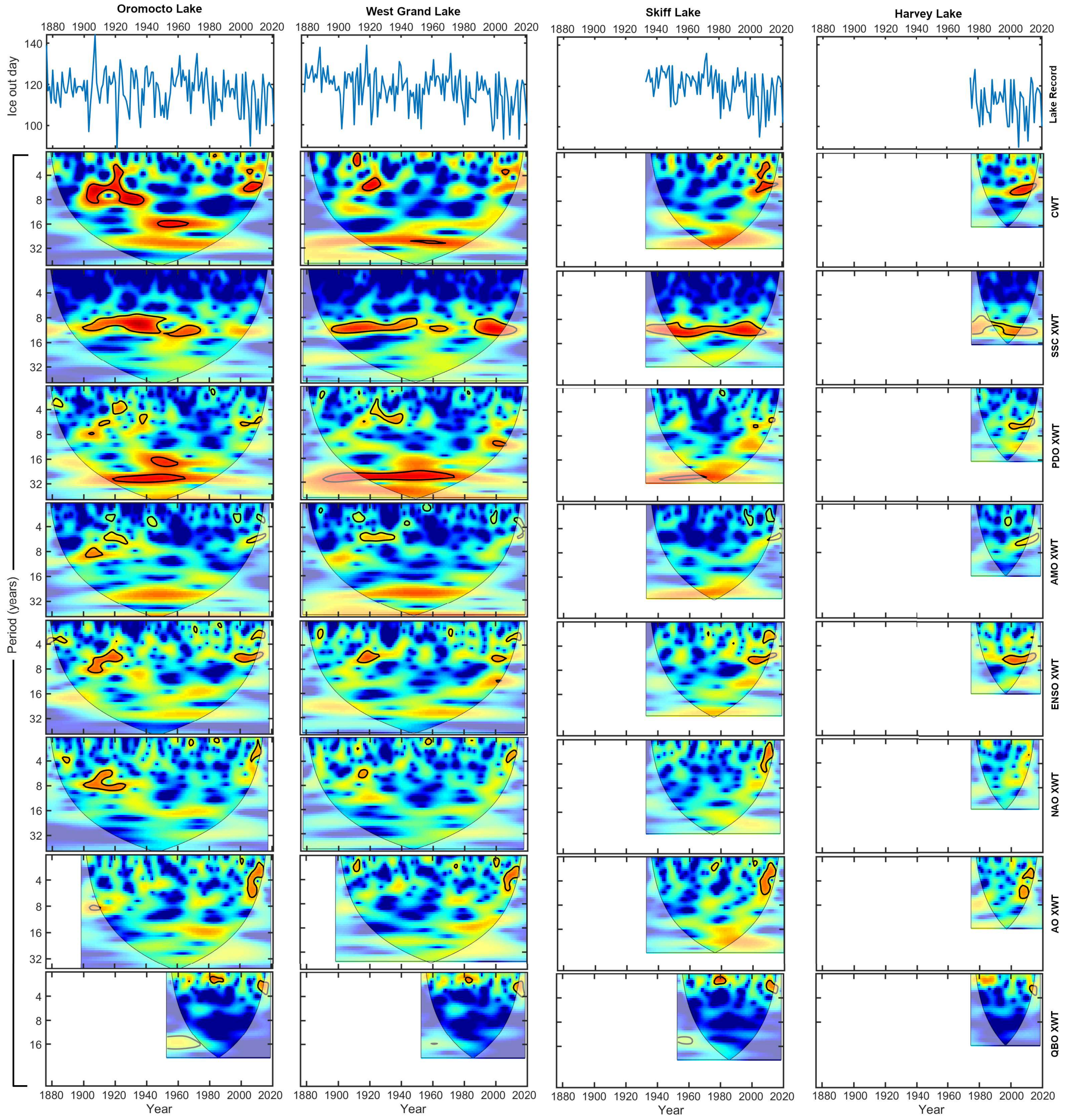

Figure S1: Graphical representation and the continuous wavelet transform (CWT) for Oromocto Lake. Cross wavelet transforms (XWTs) between lake ice out records and each of the SSC, PDO, AMO, ENSO, NAO, AO, and QBO. Time scales all span 1876–2020 for easy comparisons between the CWTs and XWTs.
